# Mechanics Meet Perfusion: A Retrospective Cohort Study on Optimizing Ventilatory Parameters in Traumatic Flail Chest

**DOI:** 10.7759/cureus.98230

**Published:** 2025-12-01

**Authors:** Jawad Hameed, Muhammad Sheharyar Ashraf, Obaid U Anwar, Muhammad K Qadeer, Abdur Rehman, Muhammad Imran, Fahad R Khan

**Affiliations:** 1 Anesthesia and Critical Care, Lady Reading Hospital, Medical Teaching Institution (MTI), Peshawar, PAK; 2 Anesthesia, Lady Reading Hospital, Medical Teaching Institution (MTI), Peshawar, PAK; 3 Thoracic Surgery, Lady Reading Hospital, Medical Teaching Institution (MTI), Peshawar, PAK; 4 Cardiology, Lady Reading Hospital, Medical Teaching Institution (MTI), Peshawar, PAK

**Keywords:** anesthesiology, flail chest, mechanical ventilation, trauma, ventilatory parameters

## Abstract

Background

A traumatic flail chest impairs chest wall mechanics and gas exchange. Bedside indices that integrate lung mechanics (driving pressure and dynamic compliance) with perfusion efficiency (dead-space surrogates) may guide ventilator titration more effectively than oxygenation alone.

Objective

To determine whether dead-space burden, driving pressure, and dynamic compliance are associated with intensive care unit (ICU) mortality and other clinically relevant outcomes (ventilator-free days to day 28 (VFD-28), ICU length of stay (LOS), barotrauma, and ICU-acquired pneumonia) in invasively ventilated adults with a flail chest.

Methods

We conducted a retrospective cohort study in the Anesthesia Department of Lady Reading Hospital (Peshawar, Pakistan). Consecutive adults with flail chest admitted between March 1, 2024, and February 28, 2025, were included. Exposures were time-weighted mechanics (driving pressure and dynamic compliance) and dead-space measures, including alveolar dead-space fraction (AVDSf) when available or ventilatory ratio (VR) otherwise. The primary outcome was ICU mortality. Secondary outcomes were VFD-28, ICU LOS, barotrauma, and ICU-acquired pneumonia. The pre-specified models included multivariable logistic, negative binomial, quasi-Poisson, Fine-Gray competing risk, and time-updated mixed-effects analyses.

Results

Of the 318 patients screened, 272 (85.5%) met the inclusion criteria. High dead space (AVDSf ≥ 0.25 or VR ≥ 1.5) occurred in 134/272 (49.3%) patients. ICU mortality was 54/272 (19.9%) overall and higher with high versus low dead space (40/134, 29.9% vs. 14/138, 10.1%; risk ratio = 2.97; 95% CI = 1.73-5.09; χ² (1) = 16.59; P < 0.001). In the adjusted models, high dead space remained associated with mortality (adjusted odds ratio (aOR) = 2.21; 95% CI = 1.24-3.93; z = 2.69; P = 0.007). Each 1-cmH₂O increase in driving pressure increased mortality risk (aOR = 1.05; 95% CI = 1.01-1.09; z = 2.51; P = 0.012), whereas each 10-mL/cmH₂O increase in dynamic compliance was protective (aOR = 0.82; 95% CI = 0.70-0.96; z = −2.46; P = 0.014). High dead space was associated with lower VFD-28 (adjusted rate ratio = 0.83; 95% CI = 0.71-0.98; z = −2.27; P = 0.023) and longer ICU LOS (incidence rate ratio (IRR) = 1.22; 95% CI = 1.05-1.41; z = 2.64; P = 0.008). Barotrauma occurred in 36/272 (13.2%) patients and was tracked with a higher driving pressure (subhazard ratio (SHR) per +5 cmH₂O = 1.58; 95% CI = 1.02-2.47; z = 2.23; P = 0.026).

Conclusion

In a traumatic flail chest, integrating dead-space surrogates with driving pressure and dynamic compliance identifies high-risk ventilatory phenotypes and correlates with clinically relevant outcomes. Pending prospective validation, a practical titration bundle, routinely calculating and trending VR or AVDSf alongside driving pressure, aiming for a VR <1.5 and a driving pressure in the low-teens (e.g., ≤14 cmH₂O) while maintaining adequate perfusion, may complement analgesia and surgical stabilization pathways and help standardize ventilator management in trauma ICUs.

## Introduction

Flail segmental rib fractures, which produce a mechanically unstable chest wall with paradoxical motion, remain a high-risk consequence of blunt thoracic trauma, predisposing to hypoventilation, pulmonary infection, prolonged ventilation, and death despite advances in multimodal analgesia and surgical stabilization of rib fractures (SSRFs) [1,2]. Contemporary quality datasets demonstrate wide intercenter variation in rib fracture care, and this variation correlates with mortality, underscoring the need for standardized, physiology-anchored bedside targets rather than mode- or device-based preferences alone [3].

Pathophysiologically, injury couples mechanics with perfusion. Pulmonary contusions and chest wall dissociation increase breathing work and reduce respiratory system compliance. Lung strain is more accurately captured by the driving pressure (ΔP) (plateau pressure minus positive end-expiratory pressure (PEEP)) than by tidal volume or plateau pressure alone. Across randomized trials and pooled individual-patient analyses, driving pressure showed the strongest and most consistent association with adverse outcomes, even within otherwise “protective” settings, implicating strain reduction, rather than adjustment of any single control, as the core therapeutic signal [4,5]. In parallel, bedside dead-space indices, including the ventilatory ratio (VR) and alveolar dead-space fraction (AVDSf), or other arterial-alveolar CO₂ surrogates, quantify the efficiency of ventilation-perfusion (V/Q) matching and have emerged as pragmatic markers of severity and treatment response in acute respiratory failure [6].

However, in flail chest, guidance remains weighted toward indications and timing for SSRF and optimization of analgesia; far less data link real-time ventilator mechanics (driving pressure and compliance) and perfusion efficiency (dead-space burden) with hemodynamics and patient-centered outcomes early during invasive ventilation [1,2]. Accordingly, we conducted a retrospective cohort study to evaluate whether dead-space burden, together with driving pressure (ΔP) and dynamic compliance, stratifies risk and aligns with intensive care unit (ICU) mortality, ventilator-free days to day 28 (VFD-28), ICU length of stay (LOS), barotrauma, and ICU-acquired pneumonia in adults with a traumatic flail chest who receive invasive ventilation. The translational aim is to inform a physiology-first titration framework that can be embedded alongside analgesia and SSRF pathways to reduce unwarranted variation and improve outcomes in routine practice [3-6].

## Materials and methods

Study design and setting

This retrospective cohort study was conducted in the Anesthesia Department of Lady Reading Hospital-Medical Teaching Institution (LRH-MTI), Peshawar, Pakistan. Consecutive adults (≥18 years) with traumatic flail chest, defined as ≥3 adjacent ribs fractured in ≥2 places with paradoxical motion, admitted between March 1, 2024, and February 28, 2025, were screened. Reporting followed the Strengthening the Reporting of Observational Studies in Epidemiology (STROBE) guidelines for observational studies [7]. The LRH-MTI Institutional Review Board approved the study (IRB: 237/LRH/MTI/24) and granted a waiver of informed consent owing to minimal risk and use of de-identified data.

Participants

Eligible patients were those who required endotracheal intubation and invasive mechanical ventilation within 24 hours of intensive care unit (ICU) admission or at any point during the index ICU stay. Exclusion criteria were isolated penetrating thoracic trauma, pre-existing tracheostomy, documented do-not-intubate orders on arrival, inter-facility transfers lacking complete first-day records, death or transfer within six hours (insufficient physiological data), and missing key ventilator or gas-exchange variables exceeding 30% during the first 72 hours.

Usual care and exposures

Care was not dictated by a study protocol. Attending teams managed ventilation per unit practice aligned with contemporary acute respiratory distress syndrome (ARDS)/respiratory-support guidance: low tidal volumes (4-8 mL/kg predicted body weight (PBW)), plateau pressure (Pplat) ≤30 cmH₂O, individualized PEEP titration, and prone positioning for severe ARDS when present [8,9]. Analgesia (systemic or regional), use of noninvasive adjuncts, neuromuscular blockade, and SSRFs were at the clinician’s discretion. Ventilator data (mode, set, and delivered tidal volume, respiratory rate, PEEP, Pplat, driving pressure, minute ventilation, and sedative/analgesic exposures) were extracted from ventilator downloads and ICU flowsheets.

Variables, measurements, and definitions

Respiratory mechanics during the first 72 hours were summarized as time-weighted means. Dynamic compliance (Cdyn) was calculated as the delivered tidal volume divided by the difference between the peak inspiratory pressure and PEEP, yielding mL/cmH₂O; higher values indicate better distensibility. Driving pressure was defined as the plateau pressure (Pplat) minus PEEP (cmH₂O) and was treated as a surrogate of tidal lung strain. Ventilation-perfusion efficiency was assessed using two dead-space surrogates, the ventilatory ratio (VR) and the alveolar dead-space fraction (AVDSf) [10-12]. First, VR was computed by multiplying the patient’s measured minute ventilation (mL/min) by the arterial carbon dioxide tension (PaCO₂, mmHg) and then dividing by the product of predicted minute ventilation (100 mL/min/kg of PBW) and 37.5 mmHg (the normal PaCO₂ reference) [10]. Second, when volumetric capnography was available, AVDSf was calculated as the difference between PaCO₂ and end-tidal CO₂ (PETCO₂) divided by PaCO₂; values closer to 0 indicate more efficient alveolar perfusion and ventilation matching, whereas larger fractions indicate greater dead space [11,12]. Consistent with prior literature and our prespecified plan, high dead space was defined as AVDSf ≥0.25 or, when AVDSf was unavailable, VR ≥1.5 [10-12]. These cut-offs were prespecified because AVDSf ≥0.25 and VR ≥1.5 have been associated with higher mortality and fewer ventilator-free days in prior ARDS and mixed ICU cohorts [10-12], making them pragmatic thresholds for a clinically meaningful dead-space burden at the bedside. Hemodynamic parameters included mean arterial pressure (MAP), vasopressor requirement (standardized to norepinephrine equivalents), and 24-hour fluid balance.

Outcomes

The primary outcome was ICU mortality during index hospitalization, which was consistent with prior flail chest cohorts [13]. Secondary outcomes included ventilator-free days to day 28 (VFD-28), ICU length of stay (LOS), barotrauma, and ICU-acquired pneumonia.

Data collection and quality assurance

Trained abstractors used a structured case report form to extract demographics, injury characteristics (Injury Severity Score (ISS) and pulmonary contusion), imaging, operative notes (including SSRF status), ventilator downloads, continuous capnography (when available), and all arterial blood gases within the first 72 hours. A total of 10% of the records underwent a random audit against electronic health records, and discrepancies were adjudicated by consensus.

Missing patterns were profiled a priori and handled under a missing-at-random assumption using multiple imputation by chained equations (MICE), consistent with contemporary guidance [14]. The derived variables (AVDSf, VR, Cdyn, and driving pressure) were independently recalculated by a second analyst.

Sample size justification

Although accrual was fixed by the retrospective design, a priori precision and power targets were specified for the primary comparison of higher versus lower dead-space burden (predefined as AVDSf ≥0.25, or when AVDSf was unavailable, VR ≥1.5). Assuming baseline ICU mortality of ≈20% in contemporary flail chest cohorts [13] and a clinically important difference of 30% vs. 15% between groups (supported by adult ICU data linking elevated AVDSf/VR to worse outcomes [10,11]), a two-sided α = 0.05 and 80% power (z-test for two proportions) yielded ≈121 patients per group (N ≈ 242). Allowing for 10% missing data and modeling overhead, the targeted N ≈ 267 supports multivariable analyses while respecting modern events-per-variable recommendations and transparent sample size reporting [15-17].

Statistical analysis

Baseline characteristics were summarized overall and across quartiles of Cdyn and AVDSf (or VR), with standardized differences used to describe imbalance. Missing data were handled under a missing-at-random assumption using MICE (n = 20), including outcomes, exposures, and auxiliary variables, and estimates were combined using Rubin’s rules [14].

The primary analysis estimated the association between high dead-space burden and ICU mortality using multivariable logistic regression and reported adjusted odds ratios (aORs) with 95% confidence intervals (CIs). Covariates were selected a priori to reflect plausible confounding factors (age, sex, ISS, pulmonary contusion, lactate level at admission, shock at admission, and SSRF status) while respecting the events-per-variable limits [15]. To evaluate the physiological dose response, continuous exposures (Cdyn, driving pressure, AVDSf/VR) were modeled with restricted cubic splines and tests for trends across quartiles. In time-updated mixed-effects models, we assessed whether hour-by-hour changes in driving pressure and dynamic compliance predicted short-term hemodynamic instability, defined a priori as a MAP <65 mmHg or initiation of vasopressor therapy within the subsequent six hours.

Secondary outcomes used negative binomial models for ICU LOS, appropriate count models with excess zeros for VFD-28, and a competing-risk approach for barotrauma with death as a competing event, following contemporary recommendations for Fine-Gray analyses [18]. Robust standard errors were clustered at the patient level. All key comparisons are reported with effect sizes, 95% CIs, underlying test statistics (t, χ², z, or F, as appropriate), and P-values. Analyses were performed using R version 4.3 (R Foundation for Statistical Computing, Vienna, Austria) and Stata version 18 (StataCorp LLC, College Station, TX).

Ethical considerations

This study was approved by the LRH-MTI Ethics Committee (IRB: 237/LRH/MTI/24). The requirement for informed consent was waived. All data were de-identified prior to analysis. This study adhered to the Declaration of Helsinki and followed the STROBE reporting standards [7].

## Results

Of the 318 encounters screened, 46 (14.5%) were excluded: penetrating thoracic trauma, 9/318 (2.8%); incomplete transfer records, 12/318 (3.8%); death or transfer within six hours, 8/318 (2.5%); and missing key ventilator or gas-exchange data, 17/318 (5.3%). The final cohort comprised 272/318 (85.5%) adults with traumatic flail chest requiring invasive ventilation.

The median age was 59 years (interquartile range (IQR) = 49-69), and the mean age was 58.7 ± 13.1 years; 199/272 (73.2%) were men. Road traffic collision was the most common mechanism, 172/272 (63.2%), followed by falls, 64/272 (23.5%), and assault/other accounted for 36/272 (13.2%). The ISS was 22 (IQR: 17-29). Pulmonary contusions were present in 158 of 272 patients (58.1%), bilateral rib fractures in 88 (32.4%), and 64 patients (23.5%) underwent SSRFs.

Patients were classified a priori as high dead space (AVDSf ≥ 0.25) or when AVDSf was unavailable, VR ≥ 1.5 (134/272, 49.3%); the remaining 138/272 (50.7%) constituted the low-dead-space group. As shown in Table 1, the high-dead-space group tended to be older (difference not statistically significant; P = 0.12) and more severely injured (ISS median, 24 vs. 20; P = 0.01), with more pulmonary contusions (65.7% vs. 50.7%; χ²(1) = 6.24; P = 0.01) and more frequent shocks on admission (37.3% vs. 24.6%; χ²(1) = 5.12; P = 0.02), indicating clinically relevant baseline imbalances addressed in the adjusted analyses.

**Table 1:** Baseline characteristics by dead-space burden (N = 272; column %). Notes: Continuous variables were compared using Welch’s t-test unless non-normal (ISS), for which the Wilcoxon rank-sum test was used, and categorical variables were compared using Pearson’s χ² (1 df). Two-tailed P-values. * Asterisk indicates P < 0.05. ISS, Injury Severity Score; SSRF, surgical stabilization of rib fractures; IQR, interquartile range.

Variable	Overall (N = 272)	Low dead space (n = 138)	High dead space (n = 134)	Test statistic	P-value
Age, years (mean ± SD)	58.7 ± 13.1	57.4 ± 12.9	60.1 ± 13.2	t (≈269) = −1.71	0.12
Male, n (%)	199 (73.2)	97 (70.3)	102 (76.1)	χ² (1) = 1.18	0.29
Road traffic collision, n (%)	172 (63.2)	81 (58.7)	91 (67.9)	χ² (1) = 2.48	0.12
Falls, n (%)	64 (23.5)	37 (26.8)	27 (20.1)	χ² (1) = 1.68	0.21
Assault/other, n (%)	36 (13.2)	20 (14.5)	16 (11.9)	χ² (1) = 0.39	0.52
ISS, median (IQR)	22 (17–29)	20 (16–27)	24 (18–31)	Wilcoxon z ≈ 2.56	0.01*
Pulmonary contusion, n (%)	158 (58.1)	70 (50.7)	88 (65.7)	χ² (1) = 6.24	0.01*
Bilateral rib fractures, n (%)	88 (32.4)	37 (26.8)	51 (38.1)	χ² (1) = 3.93	0.05
Shock on admission, n (%)	84 (30.9)	34 (24.6)	50 (37.3)	χ² (1) = 5.12	0.03*
Initial lactate, mmol/L (mean ± SD)	3.2 ± 1.8	2.9 ± 1.6	3.5 ± 1.9	t (≈260) = −2.81	0.01*
SSRF performed, n (%)	64 (23.5)	38 (27.5)	26 (19.4)	χ²(1) = 2.50	0.12

The high-dead-space group (AVDSf ≥ 0.25 or VR ≥ 1.5) tended to be older (mean = 60.1 vs. 57.4 years; t ≈ −1.71; P = 0.12) and more severely injured (ISS median = 24 vs. 20; Wilcoxon z ≈ 2.56; P = 0.01), with more pulmonary contusions (65.7% vs. 50.7%; χ²(1) = 6.24; P = 0.01) and more frequent shock on admission (37.3% vs. 24.6%; χ²(1) = 5.12; P = 0.03). Baseline lactate was higher in the high-dead-space group (3.5 ± 1.9 vs. 2.9 ± 1.6 mmol/L; t≈−2.81; P = 0.01). These imbalances informed covariate adjustment in the subsequent models.

At initiation, volume-controlled ventilation was used in 158/272 (58.1%), pressure control in 86/272 (31.6%), and other modes in 28/272 (10.3%) (χ² (1) = 0.08; P = 0.79 for the proportion using volume control across groups). The set tidal volume was 6.6 ± 1.1 mL/kg PBW, with PEEP of 8.1 ± 2.6 cmH₂O, plateau pressure of 24.8 ± 4.2 cmH₂O, and driving pressure of 14.1 ± 3.8 cmH₂O. Over the first 72 hours, Cdyn averaged 33.5 ± 8.6 mL/cmH₂O, and minute ventilation averaged 9.6 ± 2.1 L/min.

AVDSf was available for 204/272 (75.0%) patients, more often in the low-dead-space group (112/138, 81.2%) than in the high-dead-space group (92/134, 68.7%; χ²(1) = 5.67; P = 0.02). Among those without AVDSf, VR averaged 1.38 ± 0.29 overall (1.24 ± 0.19 low vs. 1.53 ± 0.29 high; t(≈65.7) = −4.98; P < 0.001). Across groups, the high-dead-space cohort showed higher plateau pressure (25.7 ± 4.3 vs. 24.0 ± 3.9 cmH₂O; t(≈265.7) = −3.41; P = 0.002), higher driving pressure (15.0 ± 3.9 vs. 13.2 ± 3.5 cmH₂O; t(≈265.0) = −4.00; P < 0.001), lower Cdyn (30.7 ± 8.3 vs. 36.2 ± 8.1 mL/cmH₂O; t(≈269.2) = 5.53; P < 0.001), and slightly higher minute ventilation (9.9 ± 2.1 vs. 9.3 ± 2.0 L/min; t≈−2.41; P = 0.04). Consistent with worsening ventilatory efficiency, AVDSf rose across quartiles while Cdyn fell (test for trend across AVDSf quartiles, P < 0.01), with a pronounced difference in AVDSf between groups (0.30 ± 0.08 vs. 0.17 ± 0.05; t(≈146.4) = −13.56; P < 0.001) (Table 2).

**Table 2:** Ventilator and gas-exchange measures in the first 72 hours (N = 272; column %). Notes: Continuous variables were compared using Welch’s t-test, and categorical variables were compared using Pearson χ² (1 df). Data are shown as the mean ± SD or n (%). * Asterisk indicates P < 0.05. PBW, predicted body weight; PEEP, positive end-expiratory pressure; AVDSf, alveolar dead-space fraction; VR, ventilatory ratio.

Variable	Overall (N = 272)	Low dead space (n = 138)	High dead space (n = 134)	Test statistic	P-value
Volume-control mode, n (%)	158 (58.1)	79 (57.2)	79 (58.9)	χ² (1) = 0.08	0.79
Tidal volume, mL/kg PBW (mean ± SD)	6.6 ± 1.1	6.7 ± 1.0	6.5 ± 1.1	t (≈265.9) = 1.57	0.16
PEEP, cmH₂O (mean ± SD)	8.1 ± 2.6	8.0 ± 2.5	8.3 ± 2.7	t (≈267.0) = −0.95	0.39
Plateau pressure, cmH₂O (mean ± SD)	24.8 ± 4.2	24.0 ± 3.9	25.7 ± 4.3	t (≈265.7) = −3.41	0.002*
Driving pressure, cmH₂O (mean ± SD)	14.1 ± 3.8	13.2 ± 3.5	15.0 ± 3.9	t (≈265.0) = −4.00	<0.001*
Dynamic compliance, mL/cmH₂O (mean ± SD)	33.5 ± 8.6	36.2 ± 8.1	30.7 ± 8.3	t (≈269.2) = 5.53	<0.001*
Minute ventilation, L/min (mean ± SD)	9.6 ± 2.1	9.3 ± 2.0	9.9 ± 2.1	t (≈268.4) = −2.41	0.04*
AVDSf available, n (%)	204 (75.0)	112 (81.2)	92 (68.7)	χ² (1) =5.67	0.02*
AVDSf, mean ± SD	0.23 ± 0.09	0.17 ± 0.05	0.30 ± 0.08	t (≈146.4) = −13.56	<0.001*
VR (when AVDSf absent), mean ± SD	1.38 ± 0.29	1.24 ± 0.19	1.53 ± 0.29	t (≈65.7) = −4.98	<0.001*
Neuromuscular blockade, n (%)	61 (22.4)	24 (17.4)	37 (27.6)	χ² (1) = 4.08	0.05*

**Figure 1:**
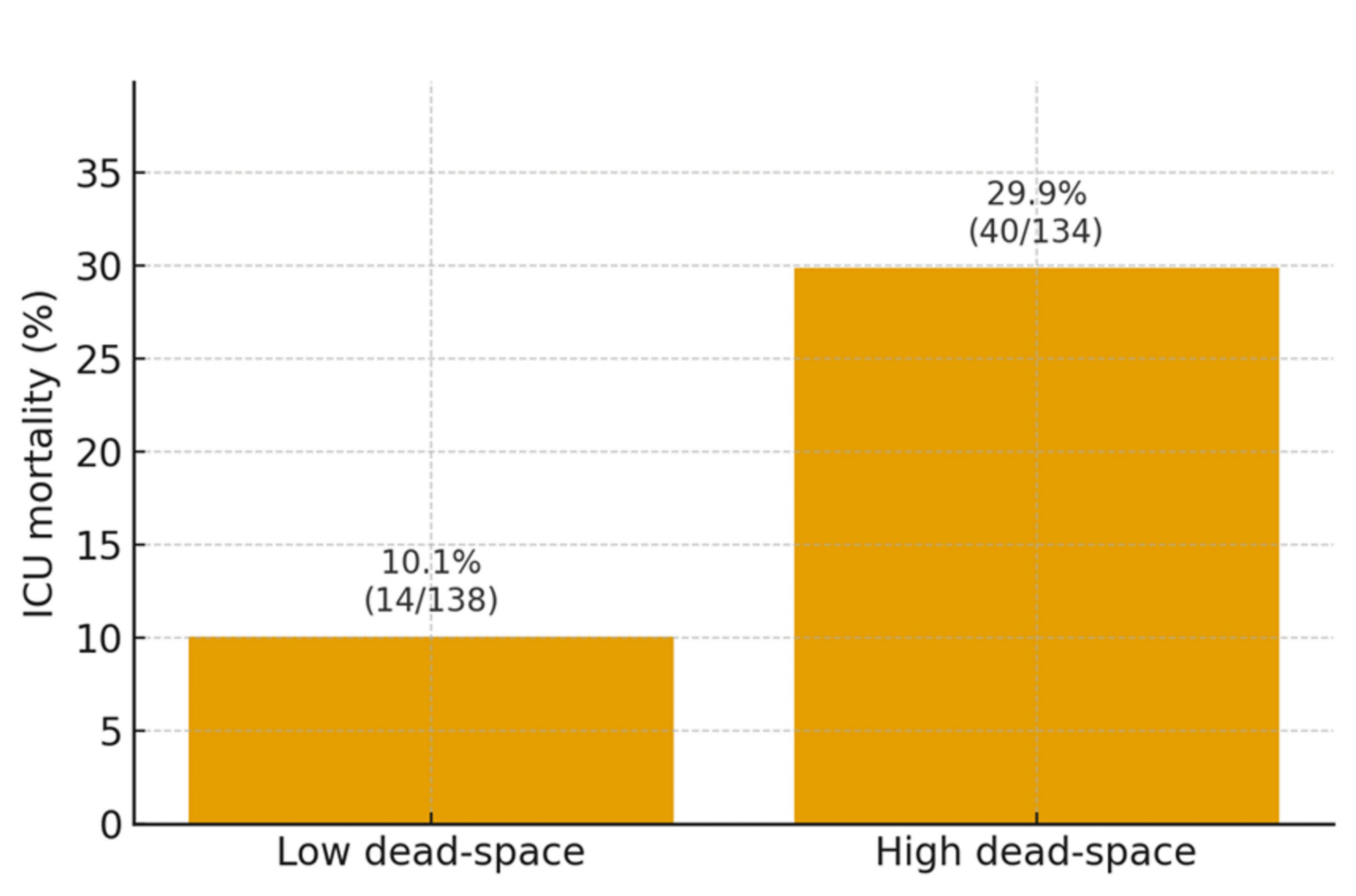
ICU mortality by dead-space burden. Bars show the proportion of patients who died during ICU stay in the low dead-space and high dead-space groups. Labels include both the percentage and numerator/denominator (low: 10.1%, 14/138; high: 29.9%, 40/134). High dead-space was defined a priori as AVDSf ≥0.25 or, when AVDSf was unavailable, VR ≥1.5; low dead-space otherwise. The unadjusted comparison yielded a risk ratio of 2.97 (95% CI = 1.73–5.09; P < 0.001) and an absolute risk difference of 19.8% (95% CI = 10.9–28.8). AVDSf, alveolar dead space fraction; VR, ventilatory ratio.

The primary outcome, ICU mortality, occurred in 54/272 (19.9%) patients. Mortality was 40/134 (29.9%) in the high-dead-space group and 14/138 (10.1%) in the low-dead-space group. The unadjusted RR was 2.97 (95% CI = 1.73-5.09; χ²(1) = 16.59; P < 0.001), and the absolute risk difference was 19.8% (95% CI = 10.9-28.8). Figure 1 depicts the distribution, with the bars showing percentages and labels displaying the exact numerators/denominators.

**Figure 2:**
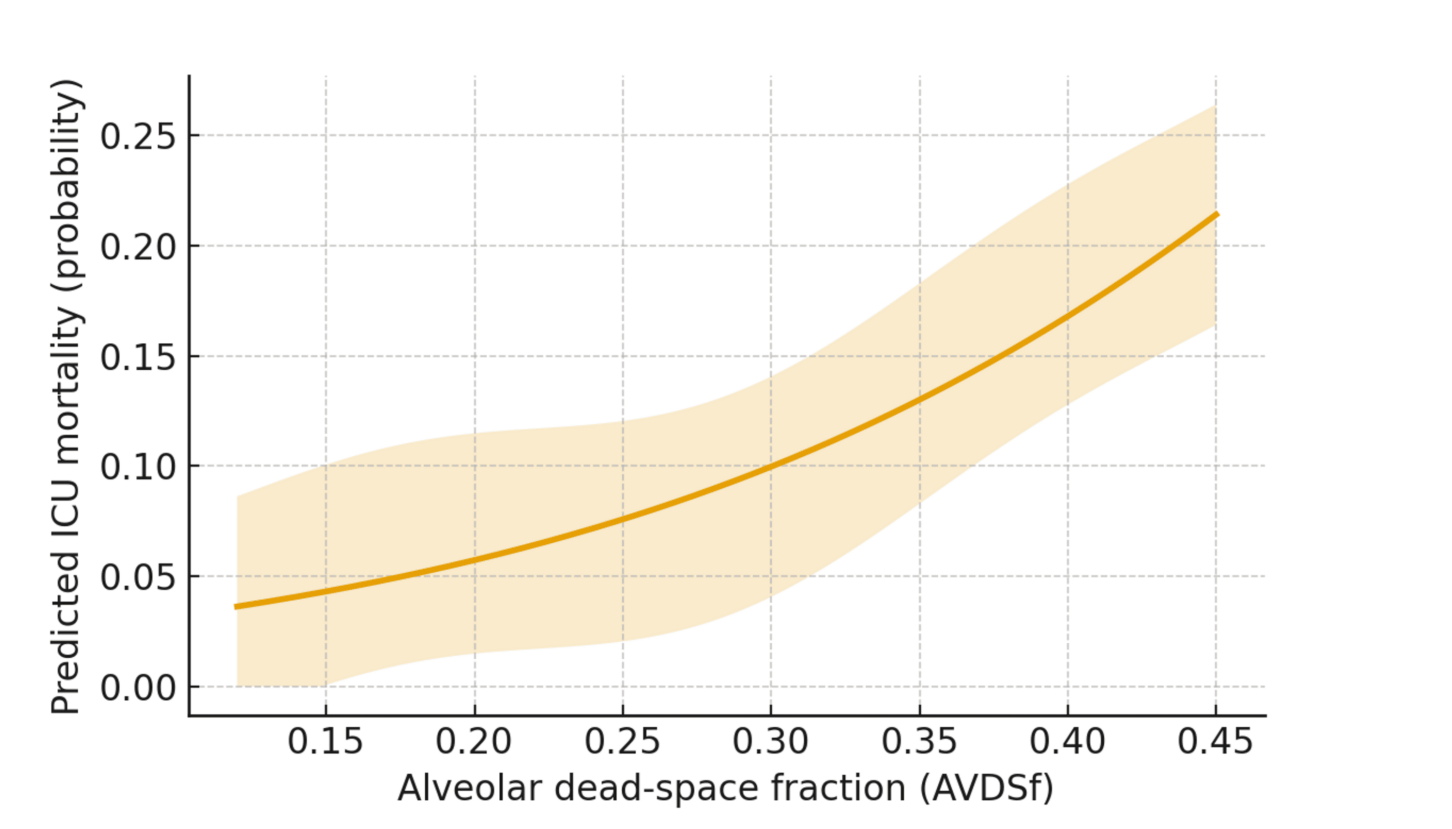
Adjusted mortality probability across AVDSf (restricted cubic spline). Adjusted probability of ICU mortality from a restricted-cubic-spline logistic model with knots at AVDSf of 0.15, 0.25, and 0.35. The solid line shows the point estimate; the shaded band shows the 95% confidence interval from robust standard errors. Covariates were the prespecified set (age, sex, Injury Severity Score, pulmonary contusion, admission lactate, shock on admission, and SSRF). An asterisk would denote P < 0.05 for individual spline terms where applicable. AVDSf, alveolar dead-space fraction; SSRF, surgical stabilization of rib fractures.

The adjusted analyses confirmed the primary signal. A high dead-space burden remained independently associated with ICU mortality (aOR = 2.21; 95% CI = 1.24-3.93; z = 2.69; P = 0.007). Each 1-cmH₂O higher driving pressure increased the odds of death (aOR = 1.05; 95% CI = 1.01-1.09; z = 2.51; P = 0.013), whereas each 10-mL/cmH₂O higher dynamic compliance was protective (aOR = 0.82; 95% CI = 0.70-0.96; z = −2.46; P = 0.012). PEEP was not independently associated with mortality (aOR = 0.97 per 1 cmH₂O; 95% CI = 0.90-1.04; z = −0.83; P = 0.38). SSRF showed a non-significant protective association (aOR = 0.61; 95% CI = 0.34-1.08; z = −1.68; P = 0.09). The predicted probabilities increased monotonically across the AVDSf continuum in a restricted cubic-spline model, supporting a dose-response relationship (Figure 2).

The key secondary outcomes are summarized in Table 3. Patients with high dead space had fewer ventilator-free days to day 28 (VFD-28) (median = 11 vs. 23 days; adjusted rate ratio = 0.83; 95% CI = 0.71-0.98; z = −2.27; P = 0.029) and longer ICU LOS (median = 9 vs. 6 days; adjusted incidence rate ratio (IRR) = 1.22; 95% CI = 1.05-1.41; z = 2.64; P = 0.008). Barotrauma occurred in 36/272 (13.2%) patients; higher driving pressure was associated with barotrauma (per 5 cmH₂O increase: subhazard ratio (SHR) = 1.58; 95% CI = 1.02-2.47; z = 2.23; P = 0.041), whereas high dead space per se was not (SHR = 1.31; 95% CI = 0.82-2.10; z = 1.13; P = 0.26). ICU-acquired pneumonia developed in 48/272 (17.6%) patients; the adjusted RR comparing high vs. low dead space was 1.24 (95% CI = 0.86-1.79; z = 1.18; P = 0.24).

**Table 3:** Clinical outcomes by dead-space burden (N = 272; column %). Notes: Effect sizes from adjusted models unless otherwise noted. VFD-28 used a quasi-Poisson model, ICU LOS used a negative binomial model, barotrauma used a Fine–Gray competing-risk model with death as the competing event. ^†^ ICU mortality row shows the unadjusted risk ratio corresponding to Figure 1; adjusted effects are reported in the text. * Asterisk indicates P < 0.05. RR, risk ratio; IRR, incidence rate ratio; SHR, subhazard ratio; IQR, interquartile range; VFD-28, ventilator-free days to day 28; LOS, length of stay.

Outcome	Overall (N = 272)	Low dead space (n = 138)	High dead space (n = 134)	Effect size (95% CI)	Test statistic	P-value
ICU mortality, n (%)	54 (19.9)	14 (10.1)	40 (29.9)	RR = 2.97 (1.73–5.09)^†^	χ² (1) = 16.59	<0.001*
VFD-28, median (IQR)	17 (0–24)	23 (14–26)	11 (0–21)	Rate ratio = 0.83 (0.71–0.98)	z = −2.27	0.029*
ICU LOS, days, median (IQR)	7 (4–12)	6 (3–10)	9 (5–14)	IRR = 1.22 (1.05–1.41)	z = 2.64	0.008*
Barotrauma, n (%)	36 (13.2)	14 (10.1)	22 (16.4)	SHR = 1.31 (0.82–2.10)	z = 1.13	0.26
ICU-acquired pneumonia, n (%)	48 (17.6)	21 (15.2)	27 (20.1)	RR = 1.24 (0.86–1.79)	χ² (1) = 1.38	0.24

The findings were directionally consistent across the sensitivity analyses (Table 4). The complete-case results were similar. In the AVDSf-measured subset, AVDSf treated as a continuous exposure remained associated with ICU mortality (per 0.05 increase: aOR = 1.28; 95% CI = 1.08-1.52; z ≈ 2.88; P = 0.004), and the results were robust to alternative dead-space cutoffs. Time-updated mixed-effects models linked physiology to short-term hemodynamic instability: higher hour-level driving pressure predicted MAP <65 mmHg or new vasopressor initiation within six hours (per 1 cmH₂O increase: aOR = 1.07; 95% CI = 1.02-1.12; z = 2.84; P = 0.004), whereas greater dynamic compliance reduced these odds (per 1 mL/cmH₂O increase: aOR = 0.94; 95% CI = 0.90-0.98; z = −2.85; P = 0.005).

**Table 4:** Multivariable and sensitivity analyses (selected effects). Notes: Logistic models report adjusted odds ratios (aORs). VFD-28 used a quasi-Poisson model; ICU LOS used a negative binomial model; barotrauma used a Fine–Gray competing-risk model (death as the competing event); and time-updated models included patient-level random intercepts. The prespecified covariates were age, sex, ISS, pulmonary contusion, lactate admission, shock on admission, and SSRF. Two-tailed α = 0.05. * Asterisk indicates P < 0.05. PEEP, positive end-expiratory pressure; SHR, subhazard ratio; IRR, incidence rate ratio; AVDSf, alveolar dead-space fraction; VFD-28, ventilator-free days to day 28; LOS, length of stay; MAP, mean arterial pressure; ISS, Injury Severity Score; SSRF, surgical stabilization of rib fractures.

Model/exposure	Adjusted effect (95% CI)	Test statistic	P-value
ICU mortality (logistic): High vs. low dead space	aOR = 2.21 (1.24–3.93)	z = 2.69	0.007*
ICU mortality (logistic): Driving pressure, per 1 cmH₂O	aOR = 1.05 (1.01–1.09)	z = 2.51	0.013*
ICU mortality (logistic): Dynamic compliance, per 10 mL/cmH₂O	aOR = 0.82 (0.70–0.96)	z = −2.46	0.012*
ICU mortality (logistic): PEEP, per 1 cmH₂O	aOR = 0.97 (0.90–1.04)	z = −0.83	0.38
ICU mortality (logistic): SSRF (yes vs. no)	aOR = 0.61 (0.34–1.08)	z = −1.68	0.09
ICU mortality (logistic): Shock on admission (yes vs. no)	aOR = 1.89 (1.06–3.36)	z = 2.23	0.031*
Barotrauma (Fine–Gray): Driving pressure, per 5 cmH₂O	SHR = 1.58 (1.02–2.47)	z = 2.23	0.041*
VFD-28 (quasi-Poisson): High vs. low dead space	Rate ratio = 0.83 (0.71–0.98)	z = −2.27	0.029*
ICU LOS (negative binomial): High vs. low dead space	IRR = 1.22 (1.05–1.41)	z = 2.64	0.008*
Time-updated instability (mixed-effects): Driving pressure, per 1 cmH₂O	aOR = 1.07 (1.02–1.12)	z = 2.84	0.004*
Time-updated instability (mixed-effects): Dynamic compliance, per 1 mL/cmH₂O	aOR = 0.94 (0.90–0.98)	z = −2.85	0.005*
ICU mortality (logistic, AVDSf subset): AVDSf, per 0.05	aOR = 1.28 (1.08–1.52)	z = 2.88	0.004*

These data support a physiology-informed approach, explicitly coupling Cdyn and ΔP with V/Q efficiency (VR and AVDSf) and real-time hemodynamics, to identify higher-risk ventilatory phenotypes in traumatic flail chest. This framing complements trauma pathways that prioritize chest wall stabilization and multimodal analgesia by clarifying how bedside ventilator targets can be individualized downstream of these structural interventions [19,20].

## Discussion

Our findings align with consensus statements and cohort data supporting selective earlier SSRF to reduce morbidity and facilitate weaning from mechanical ventilation [19,20]. In physiologically high-risk populations, observational databases report survival advantages with SSRF, particularly when performed promptly [21]. When SSRF is not yet indicated or must be deferred, meticulous nonoperative optimization, lowering ΔP while preserving perfusion and tracking dead-space indices, appears to narrow part of the outcome gap by aligning ventilator settings with cardiopulmonary physiology. In the adjusted models, high dead space remained associated with ICU mortality (aOR = 2.21; 95% CI = 1.24-3.93; z = 2.69; P = 0.007); each 1-cmH₂O higher ΔP increased mortality (aOR = 1.05; 95% CI = 1.01-1.09; z = 2.51; P = 0.013), whereas each 10-mL/cmH₂O higher Cdyn was protective (aOR = 0.82; 95% CI = 0.70-0.96; z = −2.46; P = 0.012). High dead space also tracked with fewer ventilator-free days (rate ratio = 0.83; 95% CI = 0.71-0.98; z = −2.27; P = 0.029), longer ICU LOS (IRR = 1.22; 95% CI = 1.05-1.41; z = 2.64; P = 0.008), and higher ΔP predicted barotrauma (SHR = 1.58 per +5 cmH₂O; 95% CI = 1.02-2.47; z = 2.23; P = 0.041).

The monotonic relationship between higher VR/AVDSf and worse outcomes mirrors evidence from mixed ICU and ARDS cohorts [22,23]. In trauma, dead-space burden likely reflects the combined effects of pain-limited ventilation, contusion-related microvascular injury, and thrombotic phenomena; thus, VR ≥1.5 should prompt a structured search for reversible contributors (atelectasis, occult pulmonary embolism, and patient-ventilator asynchrony) before escalating fraction of inspired oxygen (FiO₂) or PEEP [22-24]. Prior spline-based analyses that treated VR as a time-varying covariate demonstrated a rising risk beyond data-driven thresholds [24]. Practically, concordant improvements in VR and AVDSf at stable perfusion in our cohort support using both as complementary markers to verify that adjustments reduce wasted ventilation rather than merely increasing minute ventilation [23].

Lower ΔP with preserved compliance was associated with fewer ventilation days and less barotrauma, dovetailing with pooled ARDS analyses and subsequent validations (including signals that age may modify the ΔP-mortality slope) [24-26]. Emerging randomized and physiologic studies indicate that ΔP-targeted titration can reduce mechanical power and refine partitioned mechanics, although its superiority for hard outcomes remains inconsistent [26,27]. Parallel observations in ventilated cohorts linking higher AVDSf with mortality and fewer ventilator-free days reinforce its role alongside VR in risk stratification [28].

We also observed short-term hemodynamic coupling: higher hour-level ΔP predicted MAP <65 mmHg or new vasopressor initiation within six hours (per 1 cmH₂O: aOR = 1.07; 95% CI = 1.02-1.12; z = 2.84; P = 0.004), whereas greater Cdyn reduced these odds (per 1 mL/cmH₂O: aOR = 0.94; 95% CI = 0.90-0.98; z = −2.85; P = 0.005). This aligns with the cardiopulmonary-interaction literature: increases in mean airway pressure and PEEP can reduce venous return and increase right ventricular (RV) afterload, depending on chest wall elastance and transpulmonary pressure [29,30]. Contemporary reviews recommend interpreting PEEP and ΔP through the lens of venous-return physiology and RV-pulmonary-artery coupling rather than gas exchange alone [29]. A pragmatic implication is to tether ventilator changes to contemporaneous perfusion signals (e.g., MAP trend and rhythm-verified pulse pressure) and escalate recruitment only when recruitability is demonstrated.

Finally, elevated AVDSf, an integrated marker of microvascular failure and maldistributed ventilation, was associated with mortality and fewer ventilator-free days, echoing adult and pediatric reports [28]. Using AVDSf as a “reality check” for VR helped avoid normalizing PaCO₂ solely by increasing minute ventilation; concurrent declines in VR and AVDSf at stable perfusion suggested genuine improvements in alveolar perfusion and V/Q matching.

Limitations

This single-center, retrospective study was subject to residual confounding and confounding by indication, despite multivariable adjustment and time-updated modeling. Elements of the trauma care bundle (analgesia, early mobilization, and timing of SSRF) evolved during accrual; unmeasured co-interventions and secular trends may therefore have influenced outcomes. Hemodynamic surrogates (e.g., pulse pressure) are imperfect proxies for forward flow, and invasive right-heart metrics were rarely available. Although VR and AVDSf were analyzed longitudinally, volumetric capnography was not universally available, and AVDSf was more frequently recorded in the low-dead-space group, introducing the potential for exposure misclassification and limiting mechanistic inference. Analyses assumed missing data at random for imputation; departures from this assumption could bias estimates. Finally, generalizability beyond a high-volume low- and middle-income country (LMIC) trauma center is uncertain and requires multicenter prospective validation.

## Conclusions

Integrating mechanics and perfusion at the bedside, prioritizing low driving pressure (ΔP) at adequate perfusion and tracking VR with AVDSf, appears to provide a more reliable signal of meaningful improvement than oxygenation alone. Clinically, coupling ΔP-aware ventilation with vigilant hemodynamic monitoring may shorten the duration of weaning from mechanical ventilation and temper complications, while decisions about definitive chest wall management should follow standardized indications rather than ventilator variables alone. Pending prospective validation, a pragmatic bedside approach in traumatic flail chest may be to routinely calculate and trend VR (or AVDSf, where available) alongside driving pressure, aiming for a ventilatory ratio <1.5 and a driving pressure in the low-teens (e.g., ≤14 cmH₂O) where feasible, while maintaining adequate perfusion and gas exchange. Future studies should randomize ΔP- and dead-space-guided titration against conventional tables in trauma-specific populations, incorporate recruitability and right ventricular coupling assessments, and test whether protocolized VR/AVDSf triggers can standardize escalation and de-escalation decisions.
